# Chitosan-Based Nanogels: Synthesis and Toxicity Profile for Drug Delivery to Articular Joints

**DOI:** 10.3390/nano12081337

**Published:** 2022-04-13

**Authors:** Seng Manivong, Araceli Garcia Ac, Shunmoogum A. Patten, Julio C. Fernandes, Mohamed Benderdour, Xavier Banquy, Florina Moldovan, Valérie Gaëlle Roullin

**Affiliations:** 1 Centre de Recherche, CHU Sainte-Justine, Montreal, QC H3T 1C5, Canada; seng.manivong@umontreal.ca; 2 Faculté de Pharmacie, Université de Montréal, Montreal, QC H3T 1J4, Canada; araceli.ac.garcia@umontreal.ca (A.G.A.); xavier.banquy@umontreal.ca (X.B.); 3 Institut National de la Recherche Scientifique, Centre Armand-Frappier Santé Biotechnologie, Laval, QC H7V 1B7, Canada; kessen.patten@inrs.ca; 4 Centre de Recherche, Hôpital du Sacré-Cœur de Montréal, Faculté de Médecine, Université de Montréal, Montreal, QC H4J 1C5, Canada; julio.c.fernandes@umontreal.ca (J.C.F.); mohamed.benderdour@umontreal.ca (M.B.); 5 Faculté de Médecine Dentaire, Université de Montréal, Montreal, QC H3T 1J4, Canada

**Keywords:** nanogel, biopolymer, hyaluronic acid, osteoarthritis, biocompatibility, cartilage, osteocartilaginous cells

## Abstract

One important challenge in treating avascular-degraded cartilage is the development of new drugs for both pain management and joint preservation. Considerable efforts have been invested in developing nanosystems using biomaterials, such as chitosan, a widely used natural polymer exhibiting numerous advantages, i.e., non-toxic, biocompatible and biodegradable. However, even if chitosan is generally recognized as safe, the safety and biocompatibility of such nanomaterials must be addressed because of potential for greater interactions between nanomaterials and biological systems. Here, we developed chitosan-based nanogels as drug-delivery platforms and established an initial biological risk assessment for osteocartilaginous applications. We investigated the influence of synthesis parameters on the physicochemical characteristics of the resulting nanogels and their potential impact on the biocompatibility on all types of human osteocartilaginous cells. Monodisperse nanogels were synthesized with sizes ranging from 268 to 382 nm according to the acidic solution used (i.e., either citric or acetic acid) with overall positive charge surface. Our results demonstrated that purified chitosan-based nanogels neither affected cell proliferation nor induced nitric oxide production in vitro. However, nanogels were moderately genotoxic in a dose-dependent manner but did not significantly induce acute embryotoxicity in zebrafish embryos, up to 100 µg∙mL^−1^. These encouraging results hold great promise for the intra-articular delivery of drugs or diagnostic agents for joint pathologies.

## 1. Introduction

Treating joint diseases remains a challenging clinical goal despite major research efforts. Different tissues and cell types forming the joint contribute to distinct disease pathogeneses, which can either be primarily inflammatory (rheumatoid arthritis for instance), degenerative (as in chondrosarcoma) or both (such as in osteoarthritis). Unfortunately, there are few clinical cures for such pathologies to date and pharmacological treatments are mainly symptomatic, using analgesics and anti-inflammatory drugs until surgical intervention is necessary [1]. Because cartilage, the central component of any joint, is an avascular tissue [2], one drawback of such treatments is the poor control of systemic drug delivery [3], which must be improved to develop more efficient and robust therapeutic approaches for joint treatments.

Intra-articular (IA) injection is a direct delivery approach commonly used to overcome the poor joint bioavailability and allows lower dose administration of drugs, lower systemic diffusion and, consequently, less systemic side-effects [3,4,5].

However, rapid clearance of drugs from the joint via blood vessels and lymphatic system of the synovial tissue is a major limitation of IA injections [3,5,6,7]. For instance, even though IA administration of corticosteroids is appropriate to treat inflammatory joint conditions according to the OARSI guidelines [8], it shows mild to minor effects after 4 weeks post-injection, due to the short half-life of the drug in the synovial cavity, and is therefore mostly used for short-term treatment [5,9].

Nanoparticles (NPs) could address this challenge. Indeed, NPs can be used as nanocarrier systems to deliver active components to target tissues in a precise and controlled manner [10]. Moreover, nano-size materials have the ability to penetrate the extra-cellular matrix (ECM) and can be tuned to regulate their elimination [3]. Thus, one goal of developing NPs for joint treatment is to provide a local sustained release of drugs and to avoid repeated injections [3,5]. Indeed, depending on their design and physicochemical nature, these nanoparticles could be retained in the joint from 2 to 4 weeks in vivo [11,12].

Nanohydrogels, or nanogels (NGs), a subtype of polymeric nanoparticles, combine the characteristics of hydrogels (i.e., high-water content), a biopolymeric matrix mimicking the extracellular environment of osteo-cartilage tissues, possible cellular adhesion, a high loading capacity [13,14,15], and the possibility to be nanoengineered with the advantages aforementioned. Numerous NGs have been developed and can be classified into two groups according to the nature of the polymer: (i) synthetic polymers, such as the widely used polyethylene glycol (PEG); (ii) natural biopolymers including alginate, collagen, chitosan (CH) or hyaluronic acid (HA). The latter exhibit many advantages due to the fact of their biological activities [16,17,18]. HA is an essential component of the cartilage, responsible for the rheological properties of the synovial fluid, enabling it to act as a lubricant and shock absorber, and it is therefore used in visco-supplementation treatments [19,20]. Chitosan is a natural polysaccharide prepared from the deacetylation of chitin, consisting in β-(1,4)-d-glucosamine and N-acetyl-d-glucosamine units and is generally considered as a non-toxic, biocompatible and biodegradable polymer [21]. Because of its similar structure to glycosaminoglycans (GAGs), CH is widely used for tissue engineering and is among the most used natural biopolymers in biomedical applications [21,22]. Nevertheless, despite the variety of NGs intended for drug delivery to the joint [23], their safety and toxicity profiles raise concerns since they are designed to be administrated for a prolonged period of time to patients [24]. Hence, both materials and synthesis techniques play crucial roles in designing suitable injectable NGs that display biocompatible, biodegradable and enhanced retention-time properties.

In this study, we developed chitosan-based nanogels and investigated the influence of the synthesis parameters on their physicochemical characteristics. Intended to act as long-lasting drug delivery platforms for osteocartilaginous applications, we further assessed the possible toxicity of the NGs in human cells (chondrocytes, synoviocytes and osteoblasts) from osteoarthritic patients and in zebrafish embryos. Our data show that chitosan-based nanogels’ synthesis parameters are important for their physicochemical characteristics and indicate that they can be suitable for osteoarthritis drug delivery.

## 2. Materials and Methods

### 2.1. Ethical Considerations

Human tissue samples were collected in accordance with the policies regarding the ethical use of human tissues for research. The protocol used in this study was approved by the Centre Hospitalier Universitaire Sainte-Justine Ethics Committee (#1985, #2252). The animal study was approved by the Centre Hospitalier Universitaire Sainte-Justine Animal Ethic Committee, Montreal, Canada (approved protocol number #644).

### 2.2. Materials

To abridge the text, all reagents used in this study, as well as consumables and equipment are listed and detailed in Tables S1 and S2, respectively.

### 2.3. Nanogel Synthesis and Characterization

#### 2.3.1. Synthesis of CH/HA Nanogels

Nanogels were prepared as follows, based on a previously reported protocol [25,26] (Figure S1): chitosan (CH, 2.5 mg∙mL^−1^) was solubilized in either an aqueous solution of citric acid (10% (*w/v*)) (NG CH–CA10) or of acetic acid (2% (*v/v*)) (NG CH–AA2 ) and magnetically stirred overnight until complete dissolution. In parallel, a solution of sodium tripolyphosphate (TPP, 1.2 mg∙mL^−1^) and 60 kDa sodium hyaluronate (HA, 0.8 mg∙mL^−1^) in water was prepared. Both solutions were filtered (0.2 µm) prior use and synthesis was performed under the most aseptic conditions possible. The anionic TPP/HA solution was then added dropwise to the cationic CH one (1:2 volume ratio), at a constant flow rate of 4.5 mL∙min^−1^ under concomitant ultrasonic (US) mixing (550 Sonic Dismembrator, Fisher Scientific, Waltham, MA, USA, power 3/12) and moderate magnetic stirring. At the end of the addition, US were maintained for an additional 60 s then magnetic stirring was maintained for another 10 min. The colloidal solution changed from colorless to turbid (characteristic Tyndall Effect).

#### 2.3.2. Nanogel Purification and Lyophilization

Nanogel suspensions were dialyzed (Spectrum, New Brunswick, NJ, USA, Spectra/Por^®^ 6.0, MWCO 25 kDa) three times at room temperature against ×100 volumes of MilliQ ultrapure water, for 36 h, under moderate magnetic stirring in order to remove solubilizing agents (citric acid, acetic acid), excess TPP and unreacted polymer chains with low Mw.

Purified nanosuspensions were then lyophilized with sucrose (8% *w/v*) as a cryoprotective agent for a minimum of 24 h.

Finally, pH as well as the hydrodynamic diameters and zeta potentials were measured prior and after dialysis and also after lyophilization to evaluate the impact of each process on these parameters.

#### 2.3.3. CH/HA Nanogel Characterization

Dynamic light scattering (DLS) and electrophoretic light scattering (ELS) were used for measurement of average hydrodynamic diameters, polydispersity indexes (PdI), and ζ-(zeta) potential (ZP), respectively (Nanobrook Omni, Brookhaven, NY, USA). Each sample was analyzed in quadruplets at 20 °C at a scattering angle of 173°, pure or acidic water serving as a reference medium, according to the situation. DLS data were expressed in % intensity in order to detect the potential presence of aggregates in the nanogel suspensions.

After each step, nanosuspensions were centrifugated at 4 °C at 16,000× *g* for 90 min. Supernatants were then kept for pH measurements (Microprocessor pH meter, pH 211, Hanna instruments, Woonsocket, RI, USA).

The nanogel production yield (*PY%*) was calculated from the lyophilized pellet as follows:

$$PY\;\%=\frac{w_{pellet}-w_{sucrose}}{w_{HA}+w_{CH}+w_{TPP}}\times 100\;$$

where *w_pellet_* corresponds to the weight of the pellet after lyophilization; *w_sucrose_* corresponds to the calculated weight of sucrose from the sucrose concentration; *w_HA_*, *w_CH_* and *w_TPP_* correspond to initial weights of hyaluronic acid, chitosan and tripolyphosphate introduced in the reaction mixture, respectively.

### 2.4. Cells Studies

#### 2.4.1. Culture of Chondrocytes, Synoviocytes and Osteoblasts

Human chondrocytes (CD) and synoviocytes (SYN) originated from our biobank (Dre Florina Moldovan, CHU Sainte-Justine, Montreal, QC, Canada, #2252) where cells were isolated from osteoarthritic patients undergoing total knee replacement [27,28,29], and human osteoblasts (OB) were from PromoCell (C-12720, Heidelberg, Germany). CD and SYN were grown in Dulbecco’s modified Eagle’s medium (DMEM), while OB were cultivated in DMEM/F12, all enriched with 10% fetal bovine serum (FBS), 1% penicillin/streptomycin and L-glutamine (PSG). Cells were seeded in culture flasks within a maximum of ten passages at 37 °C in a humidified atmosphere of 5% CO_2_ until they reach approximately 70% confluency.

Before each treatment, growth media were removed and replaced by DMEM or DMEM/F12, 0% FBS, 1% PSG to synchronize cells at G0. Cells were incubated for another 24 h. On the next day, cells were incubated with corresponding treatment in DMEM, or DMEM/F12 5% FBS and 1% PSG media for 24, 48 or 72 h.

#### 2.4.2. MTS and LDH Assays

Nanogel cytotoxicity was quantified using both the MTS and LDH colorimetric assays. Cells were seeded at 5–10 × 10^3^ cells per well into 96-well plates and grown as described in Section 2.4.1. Untreated cells served as the negative control (and reference) while cells treated with Triton X-100 0.1% (*v/v*) were used as positive controls. Cells were exposed up to 400 μg∙mL^−1^ (2-fold series dilution) to NG CH–AA2 and NG CH–CA10 for 24, 48 and 72 h. At predetermined timepoints, 50 μL/well of media were transferred in another 96-well plate for LDH assay and another 50 μL/well for NO quantification. The remaining media were discarded and replaced by 50 µL/well of fresh media. Depending on the assay, MTS 10% (*v/v*) and LDH 2X solutions were added to each well (volume ratio of 1:1) before incubation for 2 h. Optical density (OD) was then measured at 490 nm using a microplate reader (CLARIOstar^plus^, BMG LABTECH GmbH, Ortenberg, Germany).

For both techniques, the amount of formed formazan, (absorbance at 490 nm) is representative of the metabolic activity of viable cells and of the number of damaged/dead cells in the medium for MTS and LDH assays, respectively. Cell viability and cell death were assessed as follows:

$$Cell\;viability\;\%=\frac{OD_{sample}}{OD_{negative\;control}}\times 100$$

$$Cell\;mortality\;\%=\frac{OD_{sample}-OD_{negative\;control}}{OD_{positive\;control}}\times 100$$

where *OD_sample_* corresponds to the optical density of the cells treated with the nanogels; *OD_negative control_* corresponds to the optical density of untreated cells (control group), for which cell proliferation was considered 100%; *OD_positive control_* corresponds to the optical density of cells treated with Triton X-100 0.1% 30 min prior the assay.

Three different batches of NG CH–AA2 and of NG CH–CA10 were tested during three independent experiments (*n* = 3 independent experiments for each dose and formula in quadruplets, 1 batch per experiment).

#### 2.4.3. Nitric Oxide Quantification

Nitrite (NO_2_^−^), a stable product of nitric oxide (NO), was measured in culture supernatants using a spectrophotometric method based on the Griess reaction [30], as previously described [27]. Briefly, cell supernatants were first incubated for 5 min at room temperature with a solution of sulfanilamide 1% (*w/v*) in phosphoric acid 5% (*v/v*) and protected from light. A solution of naphtyl-ethylenediamine dihydrochloride 0.1% (*w/v*) was then added for another 10 min. Absorbance was measured at 540 nm and nitrite concentration was determined using a standard calibration curve of NaNO_2_ (0–100 μM; 0–6.99 mg∙mL^−1^).

#### 2.4.4. DNA Extraction

Cells were exposed up to 400 μg∙mL^−1^ (4-fold series dilution) to NG CH–AA2 and NG CH–CA10 for 24, 48 and 72 h. DNA was extracted from chondrocytes, synoviocytes and osteoblasts with the PureLink^TM^ Genomic DNA minikit (Invitrogen, Carlsbad, CA, USA) according to the manufacturer’s protocol. DNA was then quantified by spectrophotometry at 280 and 260 nm (Epoch BioTek, Santa Clara, CA, USA) and 100 ng of DNA were loaded into a 1.0% agarose gel and detected by electrophoresis (Bio-Rad ChemiDoc Imaging System, Hercules, CA, USA). Untreated samples were split into two groups: one served as reference (negative control group) for relative DNA quantification performed with Bio-Rad Image Lab software (version 6.1, ^©^2020, Bio-Rad Laboratories, Inc., Hercules, CA, USA), while the second one was treated with DNase prior gel loading to serve as positive control. DNA extractions were performed twice with two different batches of NG CH–AA2 and of NG CH–CA10 (*n* = 2 independent experiments for each dose and formula, 1 batch per experiment).

### 2.5. Zebrafish Embryo Assay

Wild-type zebrafish (*Danio rerio*) embryos were raised at 28.5 °C and staged as previously described [31]. Healthy embryos at 4 h post-fertilization (hpf) were transferred into the wells of a 6-well plate (*n* = 20 embryos/group). Different concentrations of chitosan-based NGs were added to the zebrafish water (0, 6.25, 12.5, 25.0, 50.0 and 100.0 μg∙mL^−1^). Fish water, untreated or with NG, was renewed after 48 h incubation with the same formulation and incubated until the end of the experiment at 96 hpf. Survival rate and hatching success were monitored every 24 h for a period of 96 h under a microscope (SMZ660, Nikon Instruments Inc, Melville, NY, USA) and malformations, such as morphological abnormalities and pericardial oedema, were noted at 96 hpf. Experiments were performed in triplicates for a total of 60 embryos for each group, and three different batches of NG CH–AA2 and of NG CH–CA10 were tested (*n* = 3 independent experiments for each dose and formula, 1 batch per experiment).

### 2.6. Statistical Analysis

Results are expressed as the means ± standard deviations, and statistical analysis was performed using GraphPad Prism^®^ version 9.0.0. (2020, GraphPad Software, San Diego, CA, USA). For the in vivo experiments, results were tested by one-way analysis of variance (ANOVA) and survival curves were obtained from Kaplan–Meier curves with log-rank test, while for the in vitro experiments, results were tested by Student’s *t*-test or one-way ANOVA. Statistically, a significant difference was considered at *p* < 0.05.

## 3. Results and Discussion

### 3.1. Nanogel Synthesis: Variability Factors and Resulting Physicochemical Properties

#### 3.1.1. Optimization of the Formulation of CH-Based NG

Chitosan-based NGs were synthesized by ionic (or ionotropic) gelation following a previously reported protocol [25,26], using a positively charged chitosan solution and a negatively charged hyaluronic acid/TPP solution.

Different molecular weights (Mw) of chitosan (CH) and hyaluronic acid (HA) were tested and hydrodynamic diameter (D_H_), polydispersity index (PdI) and zeta potential (ZP) were measured by dynamic and electrophoretic light scattering.

The hydrodynamic diameter, D_H_, increased with increasing Mw of both HA and CH, and larger NGs were obtained with medium Mw (MMW, 190–310 kDa) CH compared to low Mw (LMW, 50–190 kDa) CH (506 ± 127 vs. 299 ± 34 nm, respectively, when 500 kDa HA was used) (Figure S2a) as previously reported [32,33,34]. Higher PdI values were also observed when using MMW CH with 10 kDa (PdI ~ 0.4) and 500 kDa HA (PdI ~ 0.5) as also evidenced in other studies [35]. CH characteristics are known to influence NP size, shape and the dispersity of the nanosuspension [21]. Sreekumar et al. (2018) showed that the main parameters influencing the size of chitosan–TPP nanoparticles were the initial CH concentration, its degree of acetylation (DA) and the solvent environment of the polymer. Their study showed that the average diameter of particles increased with CH concentration from 0.1 to 5 mg∙mL^−1^ and NPs from 100 to 1200 nm with a polydispersity of 0.1–0.4 were obtained. When using 2.5 mg∙mL^−1^ of CH with 20% degree of acetylation, the average D_H_ of the particles was approximately 600 nm [36]. In our case, 60 kDa HA demonstrated smaller PdI of ~0.2–0.3, with the smallest PdI value obtained with LMW CH (Figure S2b) with an average size of 221 ± 16 nm (Figure S2a). Regarding zeta potentials, the overall results showed that positively charged NG were obtained, from +37 ± 3 mV to +47 ± 5 mV, regardless of the molecular weight of both HA and CH (Figure S2c).

We further pursued the study with LMW chitosan and assessed the impact of the dialysis purification process on the same parameters. As evidenced on Figure S3, dialysis significantly increased the D_H_ of 10, 60 and 1500 kDa HA NG by a factor of 1.2 to 1.6, as these nanogels are prone to swell in pure water [26]. The best compromise in terms of size, PdI and ζ-potential was obtained for nanogels prepared from LMW chitosan (50–190 kDa) and 60 kDa hyaluronic acid. This formulation yielded the lowest and most stable, most reproducible values of physicochemical characteristics, i.e., ~220 nm/+35 mV and a PdI around 0.22. Nanoparticles with PdI below 0.3, indicating a good homogeneity in size, and surface charge greater than 25 mV, are generally considered more stable and sufficient to prevent NP aggregation [37].

Several cryoprotective agents were also tested to determine the best agent that could maintain NG integrity after lyophilization and reconstitution (Table S3). For that purpose, NG size as well as PdI were measured before and after lyophilization, and before/after ratios were calculated. Freeze-drying is an efficient process to extend the shelf-life of parenteral products [38] and a robust way to ensure that biological assays are performed on similar batches with reduced bias incidence. For nanoparticles, the main risk is the irreversible aggregation of the particles into larger aggregates [39]. Overall results showed that both parameters were increased after reconstitution with 5–20% *w/v* trehalose and lactose (up to two-fold with lactose 20% (*w/v*)). On the other hand, sucrose 10% and glucose 20% (*w/v*) demonstrated good cryoprotective properties with no impact on size and PdI after sample reconstitution. However, due to the potential toxicity for zebrafish embryos, the highest concentrations were discarded [40]. Further refinement led to the conclusion that an 8% sucrose concentration was sufficient to ensure a satisfactory reconstitution (Table S3).

#### 3.1.2. Effect of the Acidic Solution

Chitosan-based nanogels can be easily obtained via electrostatic interactions between CH and a number of negatively-charged electrostatic crosslinkers, either small molecules such as TPP [34] or larger ones such as hyaluronate, alginate or modified dextran to name a few [41]. However, in order to occur, CH must be positively charged, which is obtained by dissolving the polymer in an acidic environment to promote the protonation of the amino groups. For that purpose, two acids are mainly considered, namely, citric or acetic acid [42].

Because size and zeta potential as well as the dissolution rates and pharmacokinetics of NGs also depend on chitosan’s characteristics, including the dissolving solvent, a series of acid concentrations was tested for both acids and the resulting NGs, prepared from LMW CH and 60 kDa HA, compared for their final physicochemical properties.

**Figure 1:**
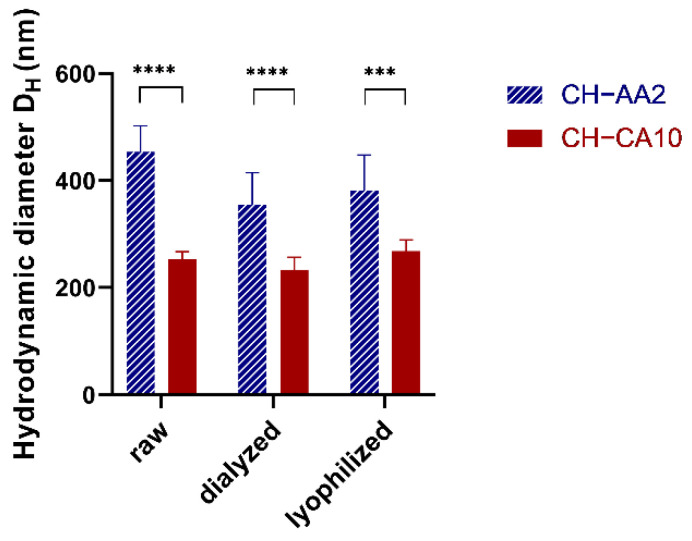
NG CH–AA2 (stripped blue) and NG CH–CA10 (red) hydrodynamic diameter DH (nm) before (raw) and after dialysis and after lyophilization and reconstitution. *n* = 3 batches (12 measurements) for NG CH–CA10 and *n* = 4 batches (16 measurements) for NG CH–AA2; *** *p* < 0.001 and **** *p* < 0.0001, Student *t*-test, GraphPad Prism version 9.0.0, GraphPad Software, CA USA.

Both NG sizes (Figure 1) and zeta potentials increased with pH as the acid concentration decreased (pH from 1.93 to 3.31, Table 1 and Table S4). 

Similar results were reported by Liu and Gao (2009) who found that nanoparticle size and zeta potential increased when the CH solution pH increased up to pH 3.5–4 and then decreased slowly until pH 5.5. However, at very low pH 1–2, CH molecules were not sufficiently cross-linked by TPP to form stable particles. One explanation was that at this critical pH value, most of the amino groups of chitosan was protonated while TPP was protonated with a lower charge density [43]. In our case, NG of 252 ± 15 nm were obtained with a pH value of the resulting nanosuspension of 1.93, indicating that both CH protonation and TPP/HA deprotonation were sufficient to form stable nanoparticles. Acetic acid 2% (*v/v*) and citric acid 10% (*w/v*) were the best compromises in terms of hydrodynamic diameters, PdI, and possibility to remove excess acid by dialysis (Table S3). Hence, for the rest of our study, LMW CH was dissolved either in acetic acid 2% (*v/v*) or in citric acid 10% (*w/v*), resulting in two formulae, namely, NG CH–AA2 and NG CH–CA10.

Gheran et al. (2018) also studied the role of the acidic environment to form gadolinium-complexed, HA/CH nanogels. Interestingly, when compared to the 10% citric acid formulation, the 10% acetic acid one resulted in smaller NG for GdDOTP CH-HA nanogels (197 vs. 242 nm) and, on the contrary, bigger NG for the GdDOTA ones (393 vs. 217 nm). However, in both cases, the acetic acid formulation led to higher PdI values (0.2 vs. 0.4), as also observed in our study [44].

Nonetheless, the fact that the quality/characteristics of the biopolymers and the fabrication process used in this study and in others were dissimilar makes a direct comparison difficult to establish across all NG formulations, as CH molecular weight, degree of acetylation, initial concentration and mass ratio of the biopolymers as well as reaction environment (pH, temperature) influence NP size, shape and dispersity of the nanosuspension [21,45].

### 3.2. Establishing Biocompatibility for CH-Based Nanogels

Cationic polymers such as chitosan or poly(ethylene imine) can exert cytotoxicity because they can aggregate on cell surfaces and interfere with intracellular activity [46,47]. Rondon et al. (2020) as previously shown that diethylaminoethyl–chitosan DEAE-CH-PEG-Folic acid/siRNA nanoparticles demonstrated overall good biocompatibilities with however, slight hemagglutination and cell damage measured by LDH regarding Raw 264.7 macrophage cells [48]. Therefore, it is of primary importance to ascertain whether a CH-based nanoformulation could serve as a drug delivery platform for osteocartilaginous applications. To do so, biocompatibility must be established, and several aspects of compatibility examined, such as cytocompatibility with the cells of interest, inflammation and genotoxicity [14]. In our case, hemocompatibility is less critical, as the NG formulations are not intended for direct blood contact but for local, intra-articular delivery.

To our knowledge, most biocompatibility studies of CH-based systems for osteocartilaginous applications only assess toxicity towards chondrocytes [49] and animal tissues [50]. However, during certain joint pathologies (osteoarthritis for instance), cartilage destruction unveils bone and leads to osteoblasts/osteoclasts exposure. In a similar manner, synoviocytes are indirectly exposed to any biomaterial introduced in the joints via the synovial liquid.

Herein, to evaluate the cytotoxicity of the two NG formulae regarding surrounding articular cartilage cells, MTS and LDH assays were performed to quantitatively estimate cell proliferation and viability of human OA chondrocytes, synoviocytes and osteoblasts after 24, 48 and 72 h incubation with 0 to 400 μg∙mL^−1^ of NG CH–CA10 or NG CH–AA2. Nitrite production was also quantified in the media, using a spectrophotometric method based on the Griess reaction, to study the effect of the NG on inflammation. Finally, potential DNA damage was evaluated after DNA extraction of the cells and embryotoxicity after exposing zebrafish embryos to NG.

#### 3.2.1. Purified CH-Based Nanogels Were Not Cytotoxic nor Generate Nitric Oxide to Osteocartilaginous Cells

**Figure 2:**
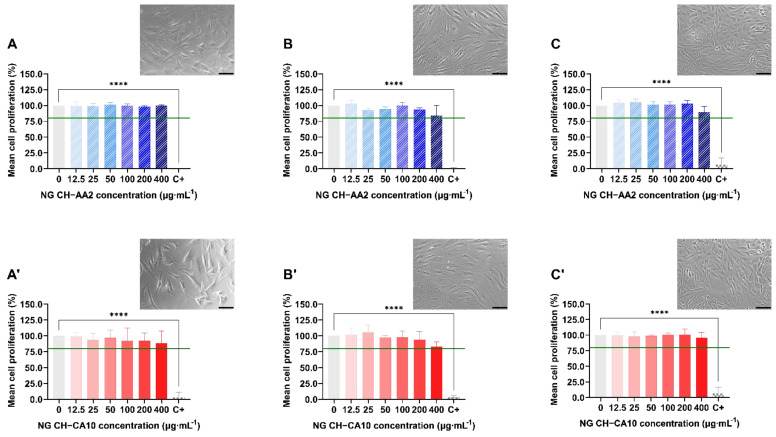
Relative proliferation and morphology of chondrocytes (**A**,**A′**), synoviocytes (**B**,**B′**) and osteoblasts (**C**,**C′**) treated with NG CH–AA2 (**A**–**C**, in stripped blue) and NG CH–CA10 (**A′**–**C′**, in full red) for 72 h. Proliferation was assessed by MTS assay and was calculated as follow: % proliferation = (*OD_sample_/OD_negative control_*) × 100. *n* = 3 independent experiments for each dose and formula in quadruplets. One-way ANOVA analyses with Dunnett’s multiple comparisons tests were performed with GraphPad Prism 9.0.0. **** *p* < 0.0001. The green line represents the lower limit of cell biocompatibility (80%). Phase contrast images (×20) of cells treated with 100 µg∙mL^−1^ at 48 h (chondrocytes and osteoblasts) and 72 h (synoviocytes). Scale bars: 100 µm.

For all three cell types, cell proliferation was superior to 80% for both NG at any concentration and any time, with no significant changes compared to the control group (Figure 2). Counterpart results from LDH assays also showed that both NG formulations maintained cellular membrane integrity and no significant mortality was observed up to a 72-h exposure (Figure 2 and Figure S4). Moreover, NG CH–AA2 and NG CH–CA10 did not induce nitric oxide (NO) production by the cells compared with the control group (Table S5), even after 72 h of treatment. NO, an inflammation product induced by IL1-beta activation, is a well-known key marker used to monitor joint inflammatory diseases [51,52,53].

Taken together, these results indicate that the synthesized CH-based NG did not induce any cellular toxicity towards chondrocytes, synoviocytes and osteoblasts, regardless of sizes and pH differences between the two formulations. This is consistent to what was previously reported for acetic acid- and citric acid-based, Gd-loaded CH-HA nanogels on rat endothelial cells, in which cell survival and membrane integrity were independent of CH concentration and of the nature of the acid used for the nanogel preparation [44]. Similarly, 100–300 nm CH/HA NG containing plasmid DNA demonstrated excellent rat synoviocytes viability (>95%) up to 80 µg∙mL^−1^ but a significant toxicity when NG concentrations reached 160 µg∙mL^−1^ [54]. Moreover, the authors noted a parallel production of prostaglandin E2 (PGE2) and NO [55]; however, it remains unclear whether the effect was attributable to the nanoparticle components, i.e., CH and HA, or to the loaded pDNA. This slight dose-dependent cytotoxicity and transitory inflammation state was also reported for CH/HA/pDNA nanoparticles in primary rabbit chondrocytes [56] and chitosan-IL-Ra and folate-chitosan-IL-Ra nanoparticles in arthritis rat osteoblasts [57]. Finally, Almalik et al. (2018) investigated the toxicological responses of CH-based NPs versus HA-coated CH-based NPs. Results from cell viability of CHO-K1 cells and ROS production assays suggested that HA coating significantly rescued cells from lethal mitochondrial injury and oxidative stress induced by CH-based NPs [58].

Therefore, the synthesized CH-based nanogels can be globally regarded as safe to osteocartilaginous cells, provided the NG concentration is not higher than 100 µg∙mL^−1^.

#### 3.2.2. Nanogels Were Moderately Genotoxic in a Dose-Dependent Manner but Did Not Significantly Induce Acute Embryotoxicity

Genotoxic effects were also assessed by DNA laddering on human chondrocytes, synoviocytes and osteoblasts treated with either 25, 100 or 400 μg∙mL^−1^ of NG CH–AA2 or NG CH–CA10 up to 72 h. In parallel, zebrafish embryos were exposed to NG up to 100 μg∙mL^−1^ from 4 to 96 hpf. Survival rates and hatching success were monitored every day and morphological abnormalities were recorded at 96 hpf.

**Figure 3:**
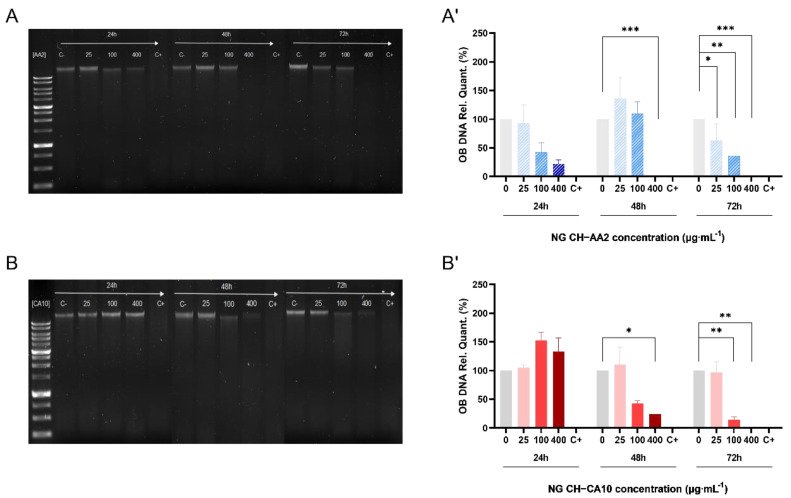
DNA degradation of osteoblasts treated with NG for 24, 48 and 72 h. One hundred nanograms of DNA were loaded into an 1.0% agarose gel when osteoblasts were treated with NG CH–AA2 (**A**) and NG CH–CA10 (**B**). DNA relative quantification of osteoblasts treated with NG CH–AA2 (**A′**) and NG CH–CA10 (**B′**) was performed using negative control bands as reference (Image Lab 6.1 Software, Bio-Rad). Experiments were performed twice for each formula and each dose. One-way ANOVA analyses with Dunnett’s multiple comparisons tests were performed with GraphPad Prism 9.0.0. * *p* < 0.05, ** *p* < 0.01, *** *p* < 0.001.

Results showed that the CH-based NGs were differently genotoxic to osteocartilaginous cells. No significant apoptotic effects of NG towards chondrocytes were noted, although synoviocyte apoptosis seemed to occur after 72 h of treatment with both types of NGs (Figures S5 and S6). Moreover, osteoblast apoptosis appeared even more rapidly, after 24 h of treatment starting from 100 μg∙mL^−1^ of NG CH–AA2 and DNA was totally degraded after 48 h exposition of 400 μg∙mL^−1^ of the same NG. This phenomenon was, however, slower for the NG CH–CA10 formulation, with DNA damage appearing with a 24-h delay compared to the NG CH–AA2, with apoptosis beginning after 48 h of 100 μg∙mL^−1^ NG (Figure 3). These results suggest that NG CH–AA2 are more toxic than NG CH–CA10 regarding osteoblasts.

Our findings are comparable to what was previously reported for the potential damage to nuclear DNA of CH-based nanogels evaluated by various techniques, such as for instance the alkaline Comet assay in SVEC4-10 murine lymph node endothelial cells [44], or TUNEL assays in Chinese hamster ovary (CHO-K1) cells [58]. In any case, similarly to our observations, data analyses showed detectable signs of DNA fragmentation only for the highest doses of CH-based nanogels. However, it is worth mentioning that cationic chitosan is able to form complexes with anionic nucleic acid through electrostatic interaction and is widely used for nonviral gene delivery [59]. Hence, increasing both polymer concentration and surface charge (zeta potential) could enhance DNA/chitosan complex formation [60] and therefore lead to low yield of DNA isolation.

To further investigate the biocompatibility of these CH-based NG and the influence of their formulation process, the toxicity of the two formulae was assessed in vivo on zebrafish embryos (*Danio rerio*). Indeed, zebrafish is a well-established model to assess biomaterial toxicity and has been proposed to serve as a high-throughput screening platform for nanotoxicity [61,62] and drug delivery [63]. Actually, zebrafish present many advantages: (i) they are highly fecund (200–300 eggs per day every 5–7 days); (ii) they grow rapidly (juvenile in 30 days and adult in 90 days); (iii) they are easy to handle (small organism); moreover, (iv) they display more than 70% homology with the human genome [61,62]. In addition, any toxicity related to the musculoskeletal development is rapidly and visually detected [64,65,66].

**Figure 4:**
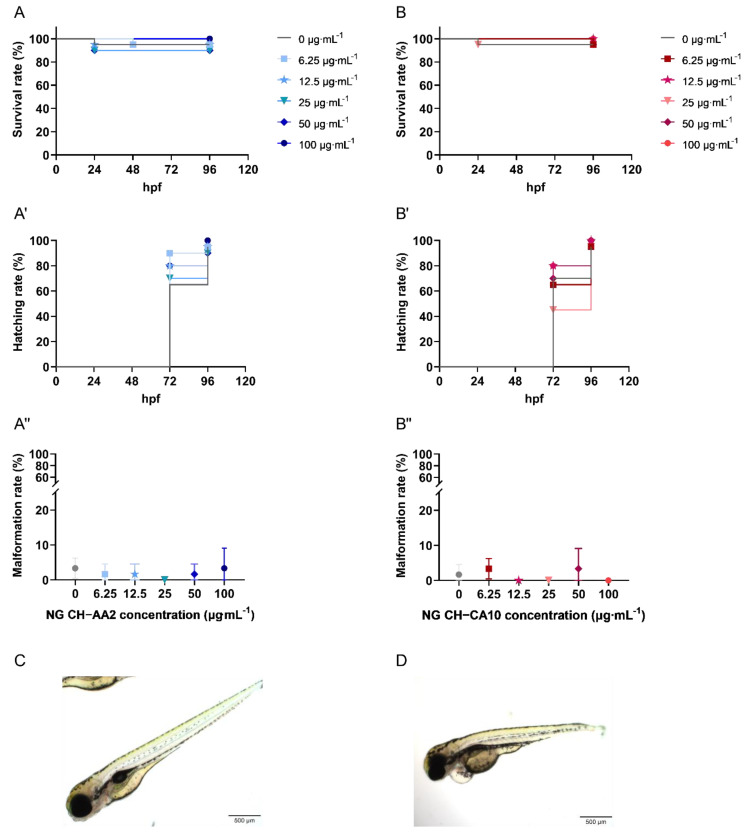
Survival, hatching and malformation rates of zebrafish (Danio rerio) embryos treated with NG CH–AA2 ((**A**, **A′**,**A″**), blue) and NG CH–CA10 ((**B**, **B′**,**B″**), red) at various concentrations. NG CH–AA2 (**A**) and NG CH–CA10 (**B**) were soaked into zebrafish water (only water for the control group) at 4 hpf. Survival and hatching were monitored every day for four days (96 h). (**C**) The gross morphology of a normal zebrafish at 96 hpf. Malformations, for example, morphological abnormalities and pericardial oedema (**D**) were recorded at the end of experiments (96 hpf). Scale bars: 100 µm (×4). *n* = 3 independent experiments per dose and per formula, including 20 embryos per dose and per test, for a total of 60 embryos per dose. Survival rates were superior to 90% and 95% for NG CH–AA2 and NG CH–CA10, respectively. No statistical difference was observed between the control and treated groups regarding neither survival nor hatching rates (%) obtained with Kaplan–Meier curves with log-rank test. The malformation rate (%) was inferior to 5% for both NG CH–AA2 and NG CH–CA10. No statistical difference was observed between control and treated groups by One-way ANOVA analyses with Dunnett’s multiple comparisons tests (GraphPad Prism 9.0.0).

Survival rates were over 90% and 95% for NG CH–AA2 and NG CH–CA10, respectively. Compared with the control group, treatment with the two NG did not significantly affect survival and hatching rates of zebrafish embryos (Figure 4). Morphological abnormalities, such as pericardial oedema and spine and tail malformations (Figure 4), were also sparingly recorded at 96 hpf. No significant differences were observed at 96 hpf compared with the control group for the two formulations at any doses (Figure 4).

Reports evaluating the toxicity of CH-based nanosystems using the zebrafish model are still scarce, which makes a direct comparison of results more complicated. For instance, Wang et al. (2016) compared the zebrafish embryonic toxicity of CH- and CH/TPP-based particles [67]. In their study, both particle types decreased the hatching rate and increased mortality in a concentration-dependent manner. Furthermore, the mortality rate of CH-based particles was higher than that CH/TPP nanogels at 120 hpf at 250 mg∙L^−1^, and more malformations were also observed when zebrafish were treated with CH-based particles (>10% at 120 hpf for 100 µg∙mL^−1^ and about 30% at 200 µg∙mL^−1^). This tendency was similarly noted by Gao et al. (2011) who reported that zebrafish embryos exposed to CH/TPP-based and ZnO nanoparticles showed a decreased in hatching rates and an increase in mortality in a concentration-dependent manner [68]. Both studies used 1% acetic acid as their CH dissolution medium, but several divergences are yet to be highlighted between these studies. First, the nanoparticle/nanogel concentrations generating toxicities in zebrafish embryos were all higher than the highest dose we tested. Nanogel sizes were also different, ranging from 85 nm (Wang et al., 2016), to 200 and 340 nm nanoparticles (Gao et al., 2011) and probably larger sizes for pure CH commercially available particles (Wang et al., 2016; average size not outlined). However, nanoparticle size seems to play a key role in zebrafish embryotoxicity, as evidenced by various studies [21,68]. Thus, interestingly, while 85 and 200 nm CH-based NPs increased the mortality rates of zebrafish embryos [67,68], no or less deaths were observed with 100–150 nm NPs and 340 nm chitosan NPs [68,69,70]. Finally, very few studies detailed how nanoparticles/nanogels were purified from their synthesis processes. A recent study demonstrated the toxicity of free low molecular weight chitosan (LMW-CH), from 12.9 to 18.5 kDa, and of LMW-CH-based nanoparticles (LMW-CH NPs). While purified LMW-CH NPs demonstrated good survival rates over 80% after 3 days, unpurified LMW-CH NPs induced rapid damage of the yolk, and later induced rapid death of zebrafish larvae in a dose dependent-manner [71]. Hence, the authors suggested that the toxicity was caused by free LMW-CH chains, notably via inducing damages to zebrafish larvae epithelium. They also investigated the influence of the fish water pH on embryo survival and showed that neither acidic double-distilled water from pH 4.0 to neutral induced animal mortality. Similar to our study, the diluted acidic NG CH–CA10 formulation demonstrated slightly higher survival rates than the NG CH–AA2 one, with survival rates of 95% and 90%, respectively, validating the fact that an initial nanosuspension of pH above 4.0 did not significantly impact zebrafish mortality. Therefore, nanosuspension with a pH range from 4.0 to 7.4 (normal joint)–7.8 (inflamed joint) [72] may be considered safe for CH-based nanogel injections, as long as the nanosuspensions have been thoroughly purified to eliminate low molecular weight free chains.

## 4. Conclusions

Our study aimed to establish an initial biological risk assessment for the use of CH-based nanogels as drug delivery platforms for osteocartilaginous applications. Although the selected biomaterials forming these NG have been separately recognized as generally safe compounds for in vivo administration, the safety and biocompatibility of such nanomaterials must be addressed because of the potential for greater interactions between nanomaterials and biological systems as stated in norm ISO 10993-22 (clause 4.4). Our data demonstrated the influence of synthesis parameters on the physicochemical characteristics of the resulting NGs and highlighted their potential impact on the biocompatibility of CH-based nanogels on all types of human, osteocartilaginous cells. Purified CH-based NGs were non-toxic neither in vitro nor in vivo in zebrafish embryos up to 100 µg∙mL^−1^. Those encouraging results hold great promise for the intra-articular delivery of drugs or diagnostic agents in otherwise poorly treated joint pathologies.

## Figures and Tables

**Table 1:** NG characterization. Mean and standard deviations of Hydrodynamic diameter D_H_ (nm) as well as Polydispersity Index (PdI), zeta potential (ZP) and pH were measured before (raw) and after dialysis and after lyophilization and reconstitution. *n* = 3 batches (12 measurements) for NG CH–CA10 and *n* = 4 batches (16 measurements) for NG CH–AA2.

	NG CH−AA2				NG CH−CA10			
	Size (nm)	PdI	ZP (mV)	pH	Size (nm)	PdI	ZP (mV)	pH
**raw**	453 ± 49	0.28 ± 0.03	49 ± 9	3.31 ± 0.02	252 ± 15	0.19 ± 0.02	27 ± 3	1.93 ± 0.02
**dialyzed**	355 ± 60	0.32 ± 0.02	40 ± 8	5.81 ± 0.09	233 ± 24	0.24 ± 0.03	30 ± 7	4.21 ± 0.12
**lyophilized**	382 ± 92	0.33 ± 0.02	40 ± 10	5.79 ± 0.11	268 ± 21	0.26 ± 0.03	27 ± 8	3.88 ± 0.54

## Data Availability

The main data supporting the results of this article are available within the paper and its Supplementary Materials. The raw and analyzed data sets generated during the study are too large to be publicly shared; however, they are available for research purposes from the corresponding authors upon reasonable request.

## Supplementary Materials

The following supporting information can be downloaded at: https://www.mdpi.com/article/10.3390/nano12081337/s1, Table S1: Detailed list of materials and reagents used in this study; Table S2: Detailed list of consumables and equipment used in this study; Table S3: Influence of the cryoprotective agent nature on the mean size and PdI of purified NG; Table S4: Physicochemical characteristics of nanogels depending on CH solubilization medium; Table S5: Nitrite production of chondrocytes, synoviocytes and osteoblasts treated with NG CH–AA2 (A) and NG CH–CA10 (B) for 72 h; Figure S1: Schematic representation of the technical setup used to synthetize chitosan-based nanogels; Figure S2: Mean sizes (a), PdI (b) and zeta potentials (c) of chitosan-based nanogels prepared by ionic gelation depending on chitosan and hyaluronic acid molecular weights. Red: low molecular weight chitosan (50–190 kDa. DDA = 77.0%); green: medium molecular weight chitosan (190–310 kDa. DDA = 83.6%); Figure S3: Mean sizes (a), PdI (b) and zeta potentials (c) of chitosan-based nanogels prepared by ionic gelation with low molecular weight chitosan, prior (red) and after (black) dialysis purification; Figure S4: Relative mortality of chondrocytes (A, A′), synoviocytes (B, B′) and osteoblasts (C, C′) treated with NG CH-AA2 (A, B, C in striped blue) and NG CH-CA10 (A′, B′, C′ in full red) for 72 h. Mortality was assessed by LDH assay and was calculated as follow: % mortality = [(*OD_sample_* − *OD_negative control_*)/*OD_positive control_*] × 100 *n* = 3 independent experiments for each dose and formula in quadruplets. One-way ANOVA analyses with Dunnett’s multiple comparisons tests were performed with GraphPad Prism 9.0.0. * *p* < 0.05. **** *p* < 0.0001. Figure S5: DNA degradation of chondrocytes treated with NG for 24 h. 48 h and 72 h. 100 ng of DNA were loaded into an 1.0% agarose gel when chondrocytes were treated with NG CH-AA2 (A) and NG CH-CA10 (B). DNA relative quantification of chondrocytes treated with NG CH-AA2 (A′) and NG CH-CA10 (B′) was performed using negative control bands as reference (Image Lab 6.1 Software. Bio-Rad). Experiments were performed twice for each formula and each dose. One-way ANOVA analyses with Dunnett’s multiple comparisons tests were performed with GraphPad Prism 9.0.0. * *p* < 0.05. ** *p* < 0.01.; Figure S6: DNA degradation of synoviocytes treated with NG for 24 h. 48 h and 72 h. 100 ng of DNA were loaded into an 1.0% agarose gel when synoviocytes were treated with NG CH-AA2 (A) and NG CH-CA10 (B). DNA relative quantification of synoviocytes treated with NG CH-AA2 (A′) and NG CH-CA10 (B′) was performed using negative control bands as reference (Image Lab 6.1 Software. Bio-Rad). Experiments were performed twice for each formula and each dose. One-way ANOVA analyses with Dunnett’s multiple comparisons tests were performed with GraphPad Prism 9.0.0. ** *p* < 0.01. *** *p* < 0.001. **** *p* < 0.0001.
