# Supplementary material for: Chitosan-Based Nanogels: Synthesis and Toxicity Profile for Drug Delivery to Articular Joints
Source: Nanomaterials (Basel). 2022 Apr 13;12(8):1337. doi: 10.3390/nano12081337 (PMC9027118; doi:10.3390/nano12081337)
Supplement: Supplementary file 1 [file nanomaterials-12-01337-s001.zip › nanomaterials-1678951-supplementary.pdf]

nanomaterials

Supplementary Materials

Chitosan-Based Nanogels: Synthesis and Toxicity Profile for Drug Delivery to Articular Joints

Seng Manivong [1,2], Araceli Garcia Ac [2], Shunmoogum A. Patten [3], Julio C. Fernandes [4], Mohamed Benderdour [4], Xavier Banquy [2], Florina Moldovan [1,5,*] and Valérie Gaëlle Roullin [2,*]

[1] Centre de recherche, CHU Sainte-Justine, Montreal, QC H3T 1C5, Canada; seng.manivong@umontreal.ca
[2] Faculté de Pharmacie, Université de Montréal, Montreal, QC H3T 1J4, Canada; araceli.ac.garcia@umontreal.ca (A.G.A.); xavier.banquy@umontreal.ca (X.B.)
[3] Institut national de la recherche scientifique, Centre Armand-Frappier Santé Biotechnologie, Laval, QC H7V 1B7, Canada; kessen.patten@inrs.ca
[4] Centre de recherche, Hôpital du Sacré-Cœur de Montréal, Faculté de Médecine, Université de Montréal, Montreal, QC H4J 1C5 Canada; julio.c.fernandes@umontreal.ca (J.C.F.); mohamed.benderdour@umontreal.ca (M.B.)
[5] Faculté de Médecine Dentaire, Université de Montréal, Montreal, QC H3T 1J4, Canada
* Correspondence: florina.moldovan@umontreal.ca (F.M.); vg.roullin@umontreal.ca (V.G.R.)

Table S1. Detailed list of materials and reagents used in this study.

Item	Acronym	Supplier	Catalogue #
**Nanogel synthesis & Characterization**			
**Item**	**Acronym**	**Supplier**	**Catalogue #**
Chitosan medium molecular weight, DDA 83.6%, viscosity 460 CPS	CS_{MMW}	Sigma-Aldrich, Missouri, USA	44887-7-50G
Chitosan low molecular weight, DDA 77%, viscosity 146 CPS	CS_{LMW}	Sigma-Aldrich, Missouri, USA	448869-50G
Chitosan low molecular weight (60-120 kDa), high purity DDA 79.1%, viscosity 27 CPS	$CS_{LMW, HP}$	Sigma-Aldrich, Missouri, USA	740063-5G
Citric acid, 99.9% grade	CA	Fisher Chemical, Thermo Fisher Scientific, MA, USA	A940-500
Acetic acid, 100% grade	AA	Fisher Chemical, Thermo Fisher Scientific, MA, USA	A940-212
Hyaluronic acid, research grade	HA1500K	LifeCore Biomedical LLC, Minnesota, USA	HA1500K
Hyaluronic acid, research grade	HA500K	LifeCore Biomedical LLC, Minnesota, USA	HA500K
Hyaluronic acid, research grade	HA60K	LifeCore Biomedical LLC, Minnesota, USA	HA60K
Hyaluronic acid, research grade	HA10K	LifeCore Biomedical LLC, Minnesota, USA	HA10K-1
Sodium TriPolyPhosphate	TPP	Alfa Aesar, MA, USA	13440-36
Sucrose 99+%	Sucrose	Sigma-Aldrich, Missouri, USA	179949-500G
Ultrapure water	–	Arium® Pro Ultrapure Water Sytem	
**Cell Culture & in vitro Assays**			
**Item**	**Acronym**	**Supplier**	**Catalogue #**
DMEM	DMEM	WISENT Inc, QC, Canada	319-165-CL
DMEM phenol red	DMEM	WISENT Inc, QC, Canada	319-005-CL
DMEM/F12	DMEM/F12	WISENT Inc, QC, Canada	319-081-CL
DMEM/F12 phenol red	DMEM/F12	WISENT Inc, QC, Canada	319-075-CL
Phosphate Buffer Saline	PBS	Fisher BioReagents	BP3994
Penicilline-Streptomycine-Glutamine	PSG	Gibco	LS10378016
Trypsine-EDTA		Invitrogen, Carlsbad, CA, USA	25200072
Fetal Bovine Serum	FBS	WISENT Inc, QC, Canada	098-150
Triton X-100 BioXtra		Sigma-Aldrich, Missouri, USA	T9284-500mL
MTS Reagent	MTS	Abcam plc. MA, USA	ab197010
LDH		Home made	
Iodonitrotetrazolium chloride	–	Sigma-Aldrich, Missouri, USA	I8377
Phenazine methosulfate	–	Santa Cruz Biotechnology, Inc., Texas, USA	sc-215700
B-nicotinamide adenine dinucleotide sodium salt	–	Sigma-Aldrich, Missouri, USA	N0632
Lithium lactate	–	Sigma-Aldrich, Missouri, USA	440469
Tris base	–	Santa Cruz Biotechnology, Inc., Texas, USA	sc-3715A
Nitric Oxid dosage	NO	Home made	
N-(1-naphtyl)ethyl-enediamine	–	Sigma-Aldrich, Missouri, USA	N-9125
Sulfanilamide	–	Sigma-Aldrich, Missouri, USA	S-9251
Sodium nitrite	–	Sigma-Aldrich, Missouri, USA	237213-100G
Phosphoric acid	–	Sigma-Aldrich, Missouri, USA	P-6560
DNA fragmentation			
PureLinkTM Genomic DNA minikit	–	Invitrogen, Carlsbad, CA, USA	K182002
Ultrapure Agarose	–	Invitrogen, Carlsbad, CA, USA	16500-500

SafeView™ Classic	–	Applied Biological Materials Inc. (abm), BC, Canada	G108
1 kb DNA Ladder RTU	–	FroggaBio, ON, Canada	DM010-R500
**In vivo Experiments**			
**Item**	**Acronym**	**Supplier**	**Catalogue #**
Adult zebrafish medium	–	Tap water	
Embryo zebrafish medium	–	Tap water with methylene blue	

Table S2. Detailed list of Consumables and Equipment used in this study.

**Nanogel synthesis & Characterization**			
**Item**	**Acronym**	**Supplier**	**Catalogue #**
Ultrasonic system Sonic Dismembrator 550	US	Fisher scientific, Thermo Fisher Scientific, MA, USA	
Ultrasonic system Sonic Dismembrator 550 probe	–	Fisher scientific, Thermo Fisher Scientific, MA, USA	FB4406
Syringe filter 5 µm Versapor® Membrane, sterile	–	PALL Corporation, Ontario, Canada	6094184
Syringe filter 0.45 µm Nylon, sterile	–	Fisher scientific, Thermo Fisher Scientific, MA, USA	13-1001-01
Syringe filter 0.20 µm Nylon, sterile	–	Fisher scientific, Thermo Fisher Scientific, MA, USA	13-1001-00
10 mL BD Luer-Lok™ Syringe, sterile	–	BD, Becton Dickinson and Company., NJ, USA	309644
BD PrecisionGlide™ Needle 25 g x 1 ½	–	BD, Becton, Dickinson and Company., NJ, USA	305127
Syringe pump, KDS-200	–	Kd Scientific, Holliston, MA, USA	78-0200
Magnetic stirrer HI 200M	–	Hanna Instruments, RI, USA	HI200M-1
Spectra/Por® Dialysis membrane 6.0, MWCO 25 kD	–	Spectrum™ Chemical Manufacturing Corp., NJ, USA	132552
Nanobrook Omni	–	Brookhaven Instruments, NY, USA	
Centrifuge 5415R	–	Eppendorf AG, Ontario, Canada	5415 R
Microprocessor pH meter	–	Hanna Instruments, RI, USA	pH 211
**Cell Culture & in vitro Assays**			
**Item**	**Acronym**	**Supplier**	**Catalogue #**
TC Flask T25, Cell+, Vented Cap	–	SARSTEDT AG & Co. KG, Nümbrecht, Germany	83.3910.302
TC Flask T75, Cell+, Vented Cap	–	SARSTEDT AG & Co. KG, Nümbrecht, Germany	83.3911.302
TC Flask T75, Stand., Vent. Cap	–	SARSTEDT AG & Co. KG, Nümbrecht, Germany	83.3911.002
6-well plate	–	CELLTREAT Scientific Products, MA, USA	229105
12-well plate	–	CELLTREAT Scientific Products, MA, USA	229111
96-well plate	–	CELLTREAT Scientific Products, MA, USA	229195
Sterile Serological Pipets, 5mL		Fisher scientific, Thermo Fisher Scientific, MA, USA	13-678-11D
Sterile Serological Pipets, 10mL	–	Greiner Bio-One GmbH	607 180
Sterile Serological pipette, 25mL		CELLTREAT Scientific Products, MA, USA	229025B

Microcentrifuges tubes 1.5mL	–	Ultident Scientific Inc., QC, Canada	87-B150-C
CLARIOstar plus microplate reader	–	BMG LABTECH GmbH, Ortenberg, Germany	
PowerPac™ Basic Power Supply	–	Bio-Rad, Hercules, CA, USA	1645050
ChemiDoc Imaging System	–	Bio-Rad, Hercules, CA, USA	
Image Lab software (version 6.1)	–	Bio-Rad, Hercules, CA, USA	
**In vivo Experiments**			
**Item**	**Acronym**	**Supplier**	**Catalogue #**
6-well plate	–	CELLTREAT Scientific Products, MA, USA	229105
Nikon SMZ660 microscope	–	Nikon Instruments Inc., NY, USA	
EVOS M5000 Imaging System	–	Thermo Fisher Scientific, MA, USA	AMF5000

Table S3. Influence of the cryoprotective agent nature on the mean size & PdI of purified NG.

	**[cryoprotective agent] %w/v**	**Blank NG**	
		**S_f/S_i**	**PdI_f/PdI_i**
Trehalose	5	1.79 ± 0.20	1.70 ± 1.29
	10	1.83 ± 1.14	1.15 ± 0.65
	20	1.16 ± 0.02	0.83 ± 0.03
Sucrose	5	1.52 ± 0.12	1.24 ± 0.36
	8	1.11 ± 0.05	1.06 ± 0.04
	10	1.00 ± 0.03	0.87 ± 0.19
	20	0.97 ± 0.00	0.57 ± 0.03
Glucose	5	2.36 ± 0.67	1.87 ± 0.52
	10	1.15 ± 0.05	1.14 ± 0.35
	20	1.01 ± 0.02	0.88 ± 0.09
Lactose	5	1.40 ± 0.03	1.21 ± 0.23
	10	1.30 ± 0.29	1.22 ± 0.18
	20	2.03 ± 1.02	2.40 ± 1.30
LMW dextran	5	1.12 ± 0.14	1.21 ± 0.52
	10	0.66 ± 0.59	Not dispersible
	20	1.12 ± 0.14	1.03 ± 0.01

Results are expressed in ratios final size after lyophilization / initial size before lyophilization (S_f/S_i) and final PdI after lyophilization / initial PdI before lyophilization (PdI_f/PdI_i). n = 3 batches, 3 measurements / batch.

Table S4. Physicochemical characteristics of nanogels depending on CH solubilization medium.

	**[acid], % *w/v***	**N***	**Size (nm)**	**PdI**	**Z-Potential (mV)**	**pH****	
			mean ± SD	mean ± SD	mean ± SD	prior	after
Citric acid (CA)	3	3	206 ± 33	0.33 ± 0.09	37.0 ± 1.4	2.4	3.5
	5	3	237 ± 15	0.37 ± 0.10	37.6 ± 2.1	2.2	3.2
	10	15	267 ± 9	0.24 ± 0.02	37.4 ± 10.1	1.3	1.9
Acetic acid (AA)	1	9	310 ± 6	0.44 ± 0.04	41.7 ± 1.9	2.6	3.6
	1.5	6	508 ± 24	0.51 ± 0.08	47.3 ± 1.5	2.7	3.4
	2	9	304 ± 21	0.38 ± 0.04	52.1 ± 0.9	1.9	3.3

* N = number of batches × 3 measurements per batch

** prior synthesis (CH-based solution) and after synthesis (NG raw suspension); average values of 2-3 measurements.

Table S5. Nitrite production of chondrocytes, synoviocytes and osteoblasts treated with NG CH-AA2 (**A**) and NG CH-CA10 (**B**) for 72 hours.

**A**		**NG CH-AA2 ($\mu g \cdot mL^{-1}$)**							
		**0**	**12,5**	**25**	**50**	**100**	**200**	**400**	**C+**
**Chondrocytes**	**[NO] (µM) (mean ± SD)**	0.2 ± 0.4	−0.2 ± 0.6	−0.2 ± 1.1	0.3 ± 0.7	1.0 ± 1.7	−0.1 ± 1.0	0.6 ± 1.6	−0.5 ± 0.4
	**Adjusted P- value**		0.9994	0.9995	0.9999	0.9511	0.9997	0.9994	0.9808
**Synoviocytes**	**[NO] (µM) (mean ± SD)**	−0.2 ± 0.2	−0.5 ± 0.2	−0.5 ± 0.4	−0.5 ± 0.2	−0.6 ± 0.2	−0.8 ± 0.4	−0.6 ± 0.5	−0.5 ± 0.3
	**Adjusted P- value**		0.8746	0.7989	0.7565	0.4873	0.1608	0.6262	0.7507
**Osteoblasts**	**[NO] (µM) (mean ± SD)**	−0.3 ± 0.9	−0.5 ± 0.4	−0.5 ± 0.2	−1.0 ± 0.2	−0.8 ± 0.2	−0.8 ± 0.6	−0.8 ± 0.4	−0.6 ± 0.1
	**Adjusted P- value**		0,9774	0,9943	0,2676	0,5934	0,635	0,5771	0,8738
**B**		**NG CH-CA10 ($\mu \cdot mL^{-1}$)**							
		**0**	**12,5**	**25**	**50**	**100**	**200**	**400**	**C+**
**Chondrocytes**	**[NO] (µM) (mean ± SD)**	0.2 ± 0.4	1.7 ± 0.1	0.7 ± 0.7	0.6 ± 0.6	1.2 ± 1.3	2.6 ± 0.6	0.7 ± 0.4	−0.5 ± 0.4
	**Adjusted P- value**		0.1756	0.9404	0.9695	0.4979	0.0303	0.938	0.8515
**Synoviocytes**	**[NO] (µM) (mean ± SD)**	−0.2 ± 0.2	0.0 ± 0.6	−0.2 ± 0.4	−0.5 ± 0.3	−0.5 ± 0.3	−0.7 ± 0.2	−0.5 ± 0.1	−0.5 ± 0.3
	**Adjusted P- value**		0.9433	>0.9999	0.8331	0.8115	0.4422	0.9117	0.7712
**Osteoblasts**	**[NO] (µM) (mean ± SD)**	−0.3 ± 0.9	−0.5 ± 0.8	−0.7 ± 0.2	−0.9 ± 0.2	−0.9 ± 0.2	−0.7 ± 0.2	−0.6 ± 0.5	−0.6 ± 0.1
	**Adjusted P- value**		0.9953	0.7546	0.5073	0.488	0.7892	0.9315	0.9082

Nitrite production was measured in the culture supernatant using a spectrophotometric method based on the Griess reaction. Absorbance was measured at 540 nm and nitrite concentration was determined using a standard solution of $NaNO_2$. n = 3 independent experiments for each dose and formula in quadruplets. One-way ANOVA analyses with Dunnett's multiple comparisons tests were performed with GraphPad Prism 9.0.0.

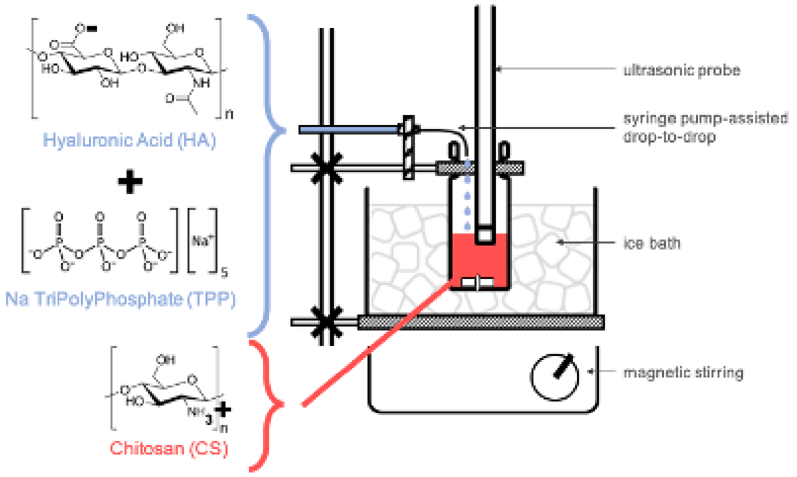

Figure S1. Schematic representation of the technical setup used to synthetize chitosan-based nanogels.

The anionic HA/TPP aqueous solution (1 vol.) is added dropwise (75 $\mu L \cdot s^{-1}$) to the cationic CS solution (2 vol.) under both moderate magnetic stirring and ultrasonic agitation. The ultrasound system consists in a Sonic Dismembrator ultrasound generator. set at power 3 out 12. equipped with a 0.5-in ultrasonic probe. The resulting solution (3 vol.) is left an additional 60 s under dual agitation. then ultrasounds are stopped and the colloidal solution is left 10 min under the sole magnetic stirring to strengthen the nanogel formation.

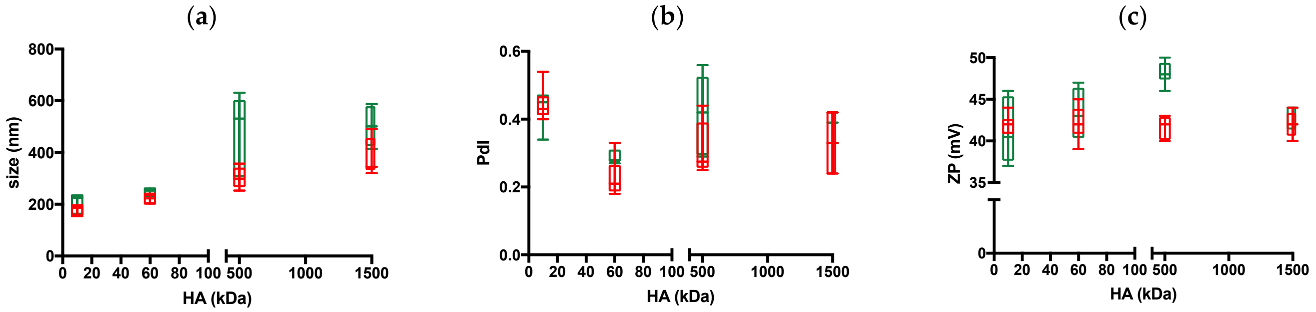

Figure S2. Mean sizes (**a**). PdI (**b**) and zeta potentials (**c**) of chitosan-based nanogels. prepared by ionic gelation depending on chitosan and hyaluronic acid molecular weights. Red: low molecular weight chitosan (50-190kDa. DDA = 77.0 %); green: medium molecular weight chitosan (190-310kDa. DDA = 83.6%).

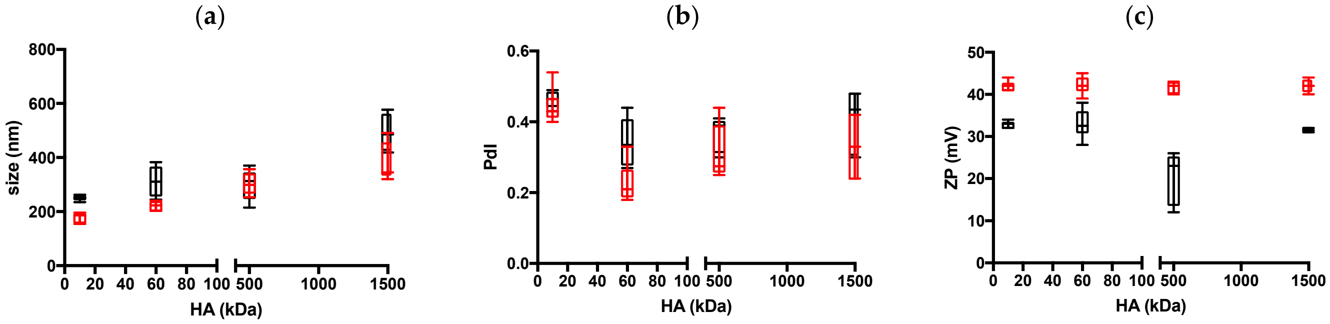

Figure S3. Mean sizes (**a**). PdI (**b**) and zeta potentials (**c**) of chitosan-based nanogels. prepared by ionic gelation with low molecular weight chitosan. prior (red) and after (black) dialysis purification.

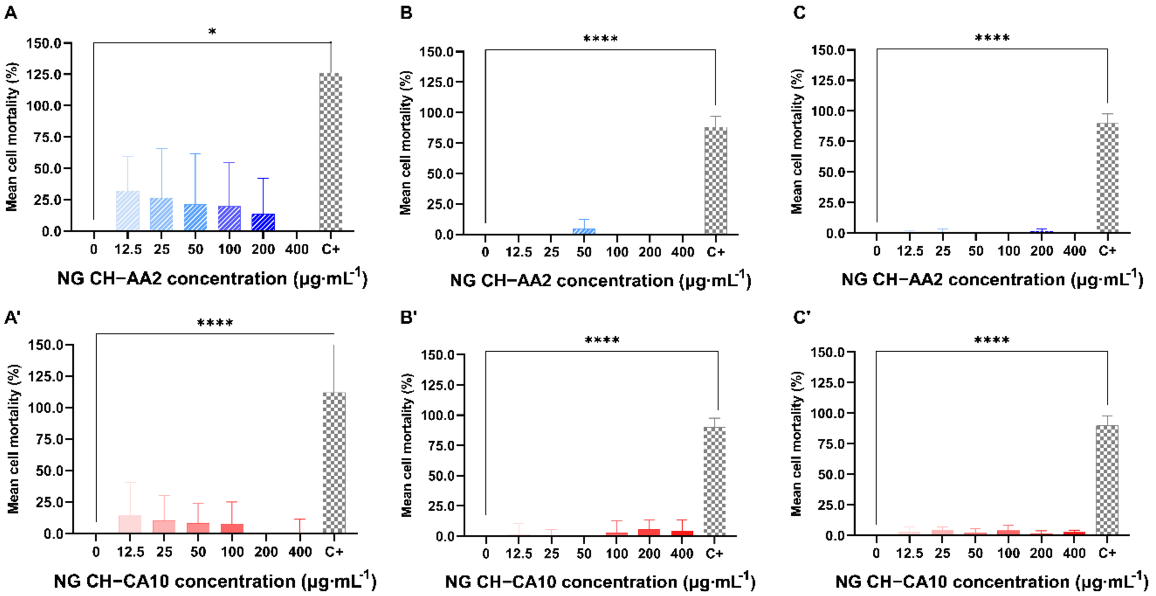

Figure S4. Relative mortality of chondrocytes (**A**,**A′**), synoviocytes (**B**,**B′**) and osteoblasts (**C**,**C′**) treated with NG CH-AA2 (**A**,**B**,**C** in striped blue) and NG CH-CA10 (**A′**,**B′**,**C′** in full red) for 72 h. Mortality was assessed by LDH assay and was calculated as follow: % mortality = $[(OD_{sample} - OD_{negative\ control})/OD_{positive\ control}] \times 100$ $n = 3$ independent experiments for each dose and formula in quadruplets. One-way ANOVA analyses with Dunnett's multiple comparisons tests were performed with GraphPad Prism 9.0.0. * $p < 0.05$. **** $p < 0.0001$.

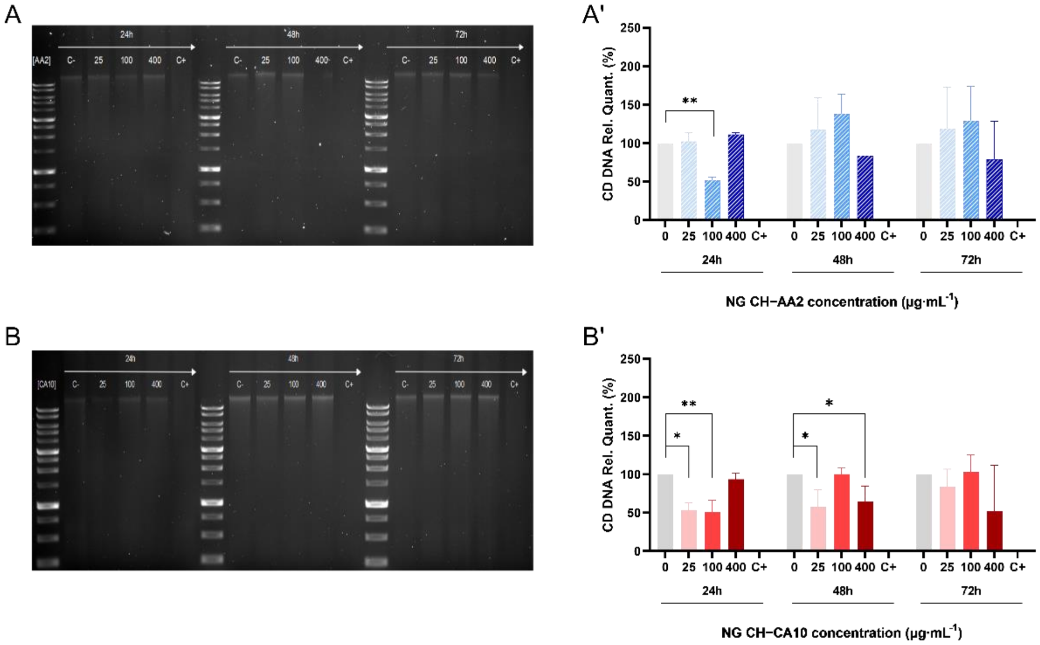

Figure S5. DNA degradation of chondrocytes treated with NG for 24 h. 48 h and 72 h. 100 ng of DNA were loaded into an 1.0% agarose gel when chondrocytes were treated with NG CH-AA2 (**A**) and NG CH-CA10 (**B**). DNA relative quantification of chondrocytes treated with NG CH-AA2 (**A′**) and NG CH-CA10 (**B′**) was performed using negative control bands as reference (Image Lab 6.1 Software. Bio-Rad). Experiments were performed twice for each formula and each dose. One-way ANOVA analyses with Dunnett's multiple comparisons tests were performed with GraphPad Prism 9.0.0. * $p < 0.05$. ** $p < 0.01$.

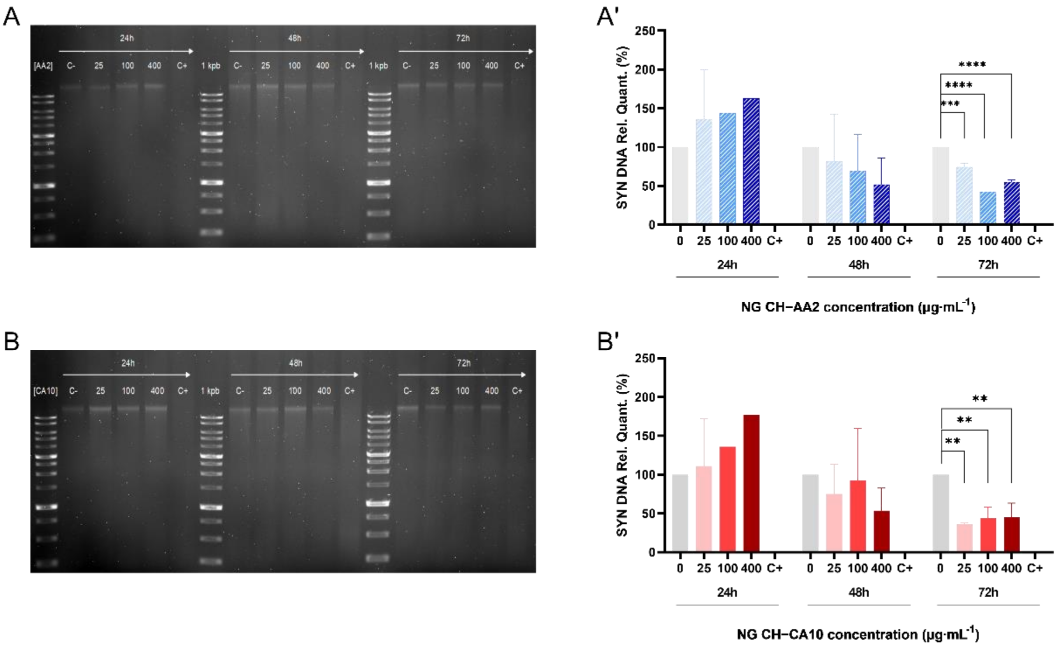

Figure S6. DNA degradation of synoviocytes treated with NG for 24 h. 48 h and 72 h. 100 ng of DNA were loaded into an 1.0% agarose gel when synoviocytes were treated with NG CH-AA2 (**A**) and NG CH-CA10 (**B**). DNA relative quantification of synoviocytes treated with NG CH-AA2 (**A′**) and NG CH-CA10 (**B′**) was performed using negative control bands as reference (Image Lab 6.1 Software. Bio-Rad). Experiments were performed twice for each formula and each dose. One-way ANOVA analyses with Dunnett's multiple comparisons tests were performed with GraphPad Prism 9.0.0. ** $p < 0.01$. *** $p < 0.001$. **** $p < 0.0001$.
